# Vasopressin V2R-Targeting Peptide Carrier Mediates siRNA Delivery into Collecting Duct Cells

**DOI:** 10.1371/journal.pone.0040010

**Published:** 2012-06-28

**Authors:** Hyun Jun Jung, Jung-Suk Lim, Hyo-Jung Choi, Mi Suk Lee, Jong-Ho Kim, Sang-Yeob Kim, Soyoun Kim, Eunjung Kim, Tae-Hwan Kwon

**Affiliations:** 1 Department of Biochemistry and Cell Biology, School of Medicine, Kyungpook National University, Taegu Korea; 2 Department of Pharmaceutical Science, College of Pharmacy, Kyung Hee University, Seoul, Korea; University of Geneva, Switzerland

## Abstract

Internalization of receptor proteins after interacting with specific ligands has been proposed to facilitate siRNA delivery into the target cells *via* receptor-mediated siRNA transduction. In this study, we demonstrated a novel method of vasopressin V2 receptor (V2R)-mediated siRNA delivery against AQP2 in primary cultured inner medullary collecting duct (IMCD) cells of rat kidney. We synthesized the dDAVP conjugated with nine D-arginines (dDAVP-9r) as a peptide carrier for siRNA delivery. The structure of synthetic peptide carrier showed two regions (i.e., ligand domain to V2R (dDAVP) and siRNA carrying domain (nine D-arginine)) bisected with a spacer of four glycines. The results revealed that 1) synthesized dDAVP-9r peptides formed a stable polyplex with siRNA; 2) siRNA/dDAVP-9r polyplex could bind to the V2R of IMCD cells and induced AQP2 phosphorylation (Ser 256); 3) siRNA/dDAVP-9r polyplex was stable in response to the wide range of different osmolalities, pH levels, or to the RNases; 4) fluorescein-labeled siRNA was delivered into V2R-expressing MDCK and LLC-PK1 cells by siRNA/dDAVP-9r polyplex, but not into the V2R-negative Cos-7 cells; and 5) AQP2-siRNA/dDAVP-9r polyplex effectively delivered siRNA into the IMCD cells, resulting in the significant decrease of protein abundance of AQP2, but not AQP4. Therefore, for the first time to our knowledge, we demonstrated that V2R-mediated siRNA delivery could be exploited to deliver specific siRNA to regulate abnormal expression of target proteins in V2R-expressing kidney cells. The methods could be potentially used *in vivo* to regulate abnormal expression of proteins associated with disease conditions in the V2R-expressing kidney cells.

## Introduction

RNA interference (RNAi) technology has been emerged as a potential therapeutic tool in gene therapy, since small interfering RNA (siRNA) targeting a specific gene could regulate abnormal expression of target proteins associated with disease conditions [1], [2]. However, cell- or tissue-type specificity of siRNA delivery is one of the major obstacles in RNAi therapeutics and hence siRNA-containing nanoparticles with high target-specificity is required to overcome the non-specific delivery in the systemic environment. Recent studies have suggested that peptide carriers, based on the receptor internalization after interaction between receptors and specific ligands, could be exploited for establishing a reliable method of specific delivery of siRNAs. Kumar and his colleagues demonstrated that peptide carrier named as RGV-nine D-arginine (RGV-9r) was able to deliver siRNA into mouse neuronal cells *via* an interaction with acetylcholine receptor of the plasma membrane [3]. RVG-9r contained nine D-arginines to form a complex with siRNA *via* an electrostatic interaction. Subramanya *et al*. also demonstrated that siRNA delivery using a peptide carrier successfully suppressed dengue viral replication and virus-induced immune responses in dengue infection [4]. Additionally, the strategy of cell-type specific siRNA delivery reported by Zhang *et al.* [5] demonstrated that siRNA tethered by folate-conjugated oligodeoxynucleotide was delivered by the interaction between folate and folate receptor.

In this study, we introduced a novel method of vasopressin V2 receptor (V2R)-mediated siRNA delivery in the kidney inner medullary collecting duct (IMCD) cells using a specific peptide ligand to V2R [i.e., 1-deamino-D-arginine vasopressin (dDAVP)]. Arginine vasopressin (AVP) is an anti-diuretic hormone, which increases osmotic water permeability in the renal collecting ducts *via* regulation of water channel protein aquaporin-2 (AQP2) [6]. Internalization of V2R into cytosol by clathrin-mediated endocytosis has been well established after ligand binding. Bouley *et al.* showed that endocytosis of the V2R was induced by vasopressin stimulation in LLC-PK1 cells expressing V2R-GFP [7] or FLAG-V2R [8], and hence it resulted in the decrease of V2R abundance through lysosomal degradation [9]. MDCK cells expressing V2R-GFP also showed V2R internalization in response to dDAVP stimulation [10].

We synthesized the dDAVP conjugated with nine D-arginines (dDAVP-9r) as a peptide carrier to deliver siRNA against AQPs into the IMCD cells of rat kidney through V2R internalization. We introduced, for the first time to our knowledge, a novel method of V2R-mediated siRNA delivery by demonstrating that 1) synthesized dDAVP-9r peptides formed as a stable polyplex with siRNA, 2) siRNA/dDAVP-9r polyplex could bind to the V2R of IMCD cells and induced AQP2 phosphorylation (Ser 256), 3) siRNA/dDAVP-9r polyplex was stable in response to the wide range of different osmolalities, pH levels, or to the RNases; and 4) siRNA/dDAVP-9r polyplex successfully delivered siRNA into the primary cultured IMCD cells, resulting in the significant decrease of protein abundance of AQP.

## Results

### Structure of dDAVP-9r Peptide and Formation of siRNA/dDAVP-9r Polyplex

**Figure 1:**
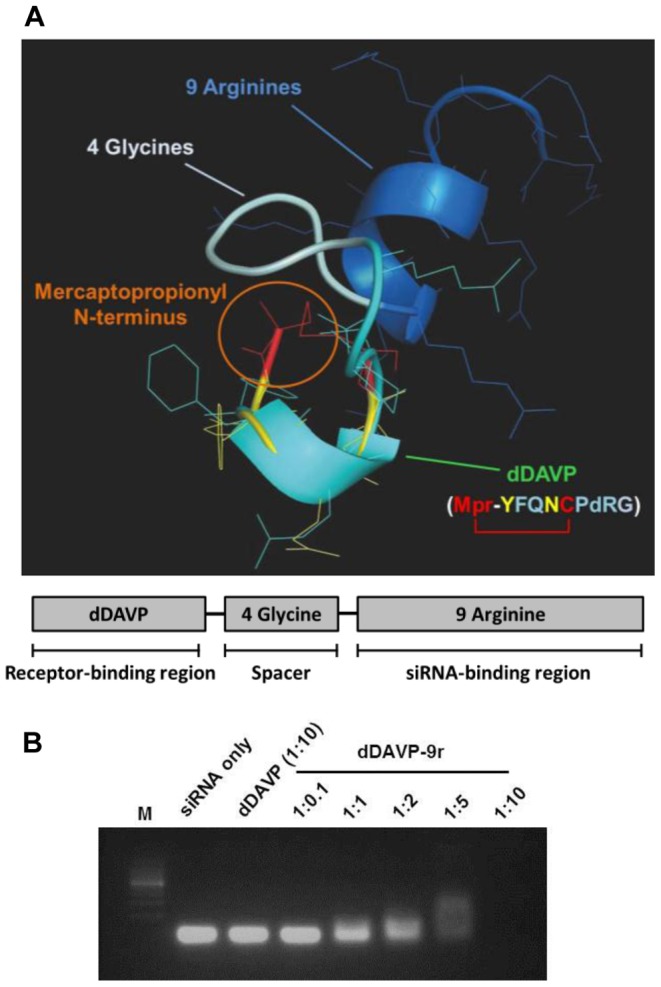
Structure of dDAVP-9r peptide and formation of siRNA/dDAVP-9r polyplex. **A**) PEP-FOLD simulations give similar structure folding of dDAVP region and vasopressin peptide. The lowest energy structure of dDAVP-4 Gly-9 Arg peptide (dDAVP, light blue; 4 Gly, gray blue; and 9 Arg, blue) was aligned with vasopressin peptide (CYFQNC, 1jk4-b, yellow). Disulfide bond of dDAVP was highlighted by red and mercaptopropionyl N-terminal modification is indicated by orange circle. **B**) Formation of siRNA/dDAVP-9r polyplex was examined at the various mole ratios by electrophoretic mobility shift assay.

dDAVP-9r peptide was composed of three domains, i.e., dDAVP (V2R binding region), four glycines (spacer), and nine D-arginines (siRNA binding region, Fig. 1A). By using the PEP-FOLD resource, the structure of dDAVP-9r peptide was investigated. The lowest energy model indicated loop conformation of dDAVP, which was followed by the helical moiety of nine-arginine stretch with a linker of four glycines. This model showed a high structural similarity of the dDAVP with the vasopressin peptide (CYFQNC, 1jk4-b, Fig. 1A), suggesting that dDAVP adopts proper folding for disulfide bond between mercaptopropionyl–N-terminus and Cys5 residue. dDAVP binds to V2R, and the domain of nine D-arginines facilitates to form a complex with siRNA molecules *via* an electrostatic interaction. Additionally, C-terminus of the peptide was modified by amidation to neutralize negative charge of C-terminus. Interaction between V2R and dDAVP may be secured from the complex of nine D-arginines and siRNA by the linker of four glycines.

To test the formation of siRNA/dDAVP-9r polyplex, electrophoretic mobility shift assay was performed. siRNA (100 pmol) was incubated with dDAVP-9r peptides at the mole ratios of 1∶0.1, 1∶1, 1∶2, 1∶5, and 1∶10 (siRNA:dDAVP-9r) in aqueous phase (PBS, pH 7.4) for 15 min, respectively. siRNA was saturated with dDAVP-9r peptides at mole ratio of 1∶10, while dDAVP-9r peptides were not made as a stable polyplex with siRNA at the mole ratio of 1∶0.1 (Fig. 1B).

### Transduction of Fluorescein-labeled siRNA in Endogenous V2R-expressing Cells Treated by FITC-siRNA/dDAVP-9r Polyplex

**Figure 2:**
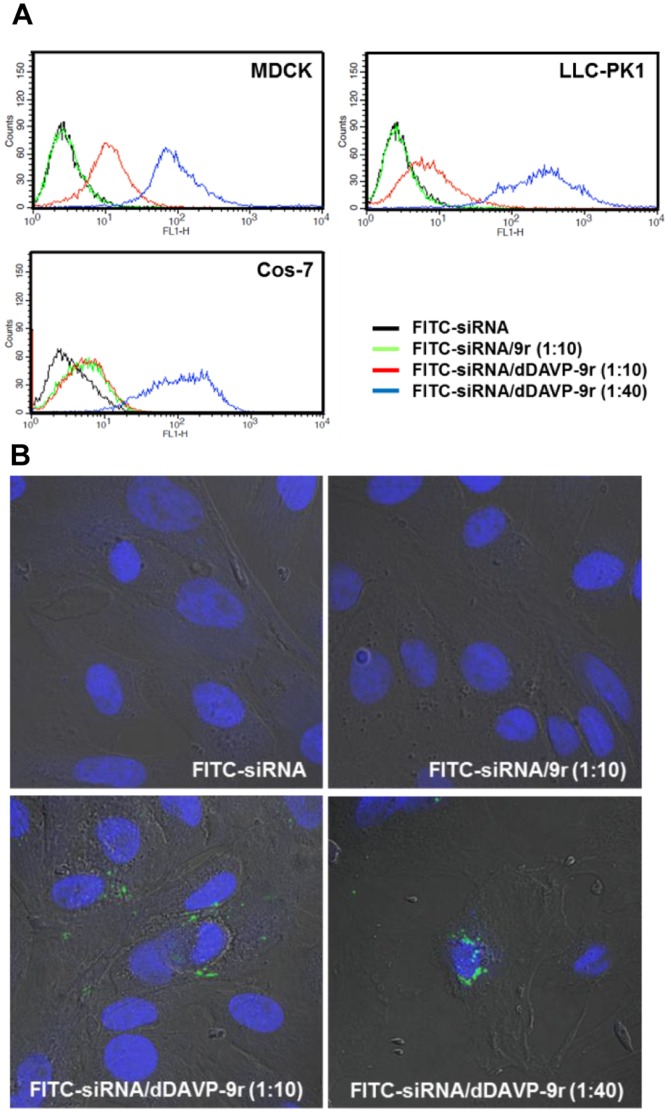
Internalization of siRNA/dDAVP-9r polyplex in V2R-expressing cells and V2R-negative cells. **A**) Cellular uptake of fluorescein-labeled siRNA in V2R-expressing MDCK cells and LLC-PK1 cells, and V2R-negative Cos-7 cells was examined by flow cytometry. Black line: FITC-siRNA alone; green line: FITC-siRNA/9r polyplex (1∶10 mole ratio, siRNA:peptide); red line: FITC-siRNA/dDAVP-9r polyplex (1∶10 mole ratio, siRNA:peptide); and blue line: FITC-siRNA/dDAVP-9r (1∶40 mole ratio, siRNA:peptide). **B**) Laser scanning confocal microscopic examination of the MDCK cells treated by FITC-labeled siRNA only or FITC-labeled siRNA complexed with 9r peptides (1∶10 mole ratio) or with dDAVP-9r peptides (1∶10 or 1∶40 mole ratio). Nuclei of the MDCK cells were stained with DAPI. All fluorescence imaging studies were performed at least three times and images demonstrated were representative of the majority of analyzed cells.

To examine the transduction of FITC-siRNA into the cells, V2R-expressing MDCK and LLC-PK1 cells [8], [11], [12] were treated by FITC-siRNA/dDAVP-9r polyplex and analyzed by flow cytometry (Fig. 2A). Cos-7 cells were also used as endogenous V2R-negative cells [8] to determine the specificity of siRNA uptake via the V2R. Cells were incubated with FITC-siRNA/dDAVP-9r polyplex at the mole ratio of 1∶10 or 1∶40 for 1 h at 37°C. In addition, FITC-siRNA alone or FITC-siRNA/9r polyplex (without dDAVP) were also tested at the mole ratio of 1∶10 (siRNA:peptide). As shown in Fig. 2A, MDCK cells and LLC-PK1 cells, which were incubated with FITC-siRNA (black line) alone or FITC-siRNA/9r polyplex (green line) exhibited much less FITC signals compared with the cells incubated with FITC-siRNA/dDAVP-9r polyplex [1∶10 mole ratio (red line) or 1∶40 mole ratio (blue line)]. Importantly, no difference in cellular uptake between FITC-siRNA/9r polyplex (1∶10 mole ratio, green line) and FITC-siRNA/dDAVP-9r polyplex (1∶10 mole ratio, red line) was found in Cos-7 cells, indicating that V2R mediated the cellular uptake of siRNA. In contrast, all the examined cell lines (MDCK, LLC-PK1, and Cos-7 cells) incubated with FITC-siRNA/dDAVP-9r polyplex of 1∶40 mole ratio showed increased cellular uptake and this could be caused non-specifically by highly positive surface charge of the polyplex, as previously demonstrated [13], [14].

Subcellular localization of the FITC-siRNA/dDAVP-9r polyplex in the MDCK cells was examined by laser scanning confocal microscopy coupled with differential interference contrast (DIC) images (Fig. 2B). Intracellular FITC signal was detected in the MDCK cells treated by FITC-siRNA/dDAVP-9r polyplex (1∶10 and 1∶40 mole ratio in Fig. 2B), whereas the FITC signal was not observed in the cells treated with FITC-siRNA alone or FITC-siRNA/9r polyplex (1∶10 mole ratio).

### Stability of siRNA/dDAVP-9r Polyplex

**Figure 3:**
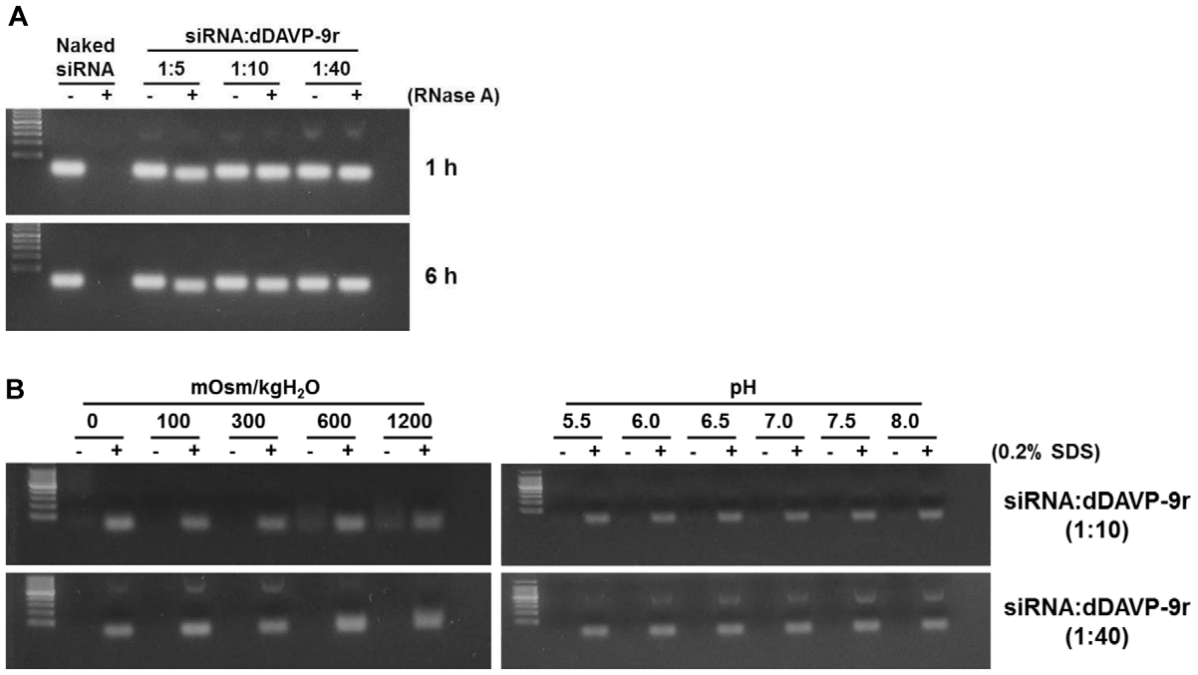
Stability of siRNA/dDAVP-9r polyplex against RNase A, osmolality, and pH. **A**) siRNA (100 pmol) or siRNA/dDAVP-9r polyplex at the various mole ratios were incubated in the absence or the presence of RNase A for 1 h or 6 h. **B**) siRNA/dDAVP-9r polyplex (1∶10 or 1∶40 mole ratio) were incubated under different osmolalities generated by NaCl and urea or different pH levels for 60 min after formation of the polyplex. The polyplex was incubated in the absence or the presence of 0.2% SDS, and siRNAs were electrophoresed on 2% agarose gel containing EtBr.

The stability of siRNA-containing carrier against ribonucleases (RNases) is required for the delivery of siRNA to the target cells or tissues *in vitro* and *in vivo* safely. To examine the resistance of siRNA/dDAVP-9r polyplex against RNases, the polyplex was incubated with 5 µg/ml RNase A for 1 h or 6 h. As shown in Fig. 3A, the naked siRNA was completely degradaded by RNase A in 1 h and 6 h incubation, whereas siRNA of the polyplex was unaffected by RNase A at the mole ratio of 1∶10 and 1∶40. In contrast, at the mole ratio of 1∶5, siRNA content was slightly decreased in the presence of RNase A (Fig. 3A).

The stability of siRNA/dDAVP-9r polyplex was also examined in response to various conditions of different osmolalities or pH levels (Fig. 3B). The polyplex was incubated in the absence or the presence of 0.2% SDS, and siRNAs were electrophoresed on 2% agarose gel containing EtBr (Fig. 3B). While at the mole ratio of 1∶40 of siRNA/dDAVP-9r polyplex the electrostatic interaction was not affected in response to different osmolalities (0, 100, 300, 600, and 1,200 mOsm/KgH_2_O), the polyplex at the mole ratio of 1∶10 was partially disturbed by the osmolality over 600 mOsm/KgH_2_O (observed at 600 and 1,200 mOsm/KgH_2_O, Fig. 3B). In contrast, the formation of siRNA/dDAVP-9r polyplex at both 1∶10 and 1∶40 was unaffected by the tested pH levels (pH 5.5, 6.0, 6.5, 7.0, 7.5, and 8, Fig. 3B). After the treatment of SDS in each lane, all the siRNA was intact (Fig. 3B).

In addition to the stability of polyplex, size and surface charge of polyplex are also important factors for the cellular uptake. Particle size of siRNA/dDAVP-9r polyplex was measured by light scattering using Zetasizer. Z-average diameters of polyplex were determined by dynamic light scattering at 25°C. siRNA/dDAVP-9r polyplex at the mole ratio of 1∶10 or 1∶40 (siRNA:dDAVP-9r) demonstrated different spectrum of particle diameters. siRNA/dDAVP-9r polyplex at 1∶10 mole ratio showed relatively constant spectrum of diameters with an average particle diameter of 204±67.7 nm. In contrast, the polyplex at 1∶40 mole ratio demonstrated wider spectrum of diameters with average particle diameter of 395.7±176.5 nm. The average particle diameter of the polyplexes was determined by triplicate measurements in two different aqueous suspension of each ratio. Additionally, zeta potential of polyplex was measured to examine the surface charge of polyplex at the mole ratio of 1∶10 and 1∶40. The values of polyplex at the mole ratio of 1∶10 were 11.13±0.38 mV, while the values of polyplex at the mole ratio of 1∶40 were 17.9±0.79 mV. Surface charge of each sample was also analyzed by triplicate measurements in two different aqueous suspension of each ratio. The results indicate that polyplex of 1∶40 mole ratio possesses highly positive surface charge, leading to non-specific interaction with negatively charged plasma membrane of cells, as previously demonstrated [13], [14].

### Interaction Between siRNA/dDAVP-9r Polyplex and V2R in IMCD Cells

dDAVP-9r peptides formed the stable polyplex with siRNAs in aqueous phase (Fig. 1). We examined whether the polyplex bind to V2R expressed at the plasma membrane of IMCD cells. To study the binding of siRNA/dDAVP-9r polyplex to V2R and their ability to stimulate V2R, IMCD tubule suspension isolated from rat kidneys were treated by dDAVP or dDAVP-9r for 3 min. Stimulation of cAMP/PKA signaling pathway by interaction between V2R and vasopressin has been known to phosphorylate AQP2 at S256 (PKA phosphorylation consensus site) [15], [16].

**Figure 4:**
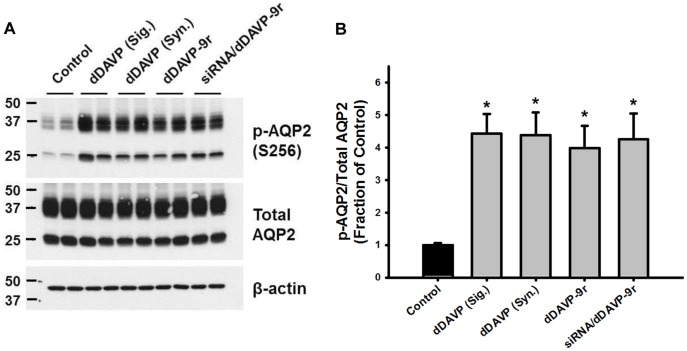
Phosphorylation of AQP2 at S256 in response to dDAVP, dDAVP-9r, and siRNA/dDAVP-9r polyplex treatment in IMCD cells of rat kidney. **A**) IMCD suspension was treated by dDAVP (10^−8^ M), dDAVP-9r peptide (10^−8^ M), or siRNA/dDAVP-9r polyplex (1∶10) for 3 min. **B**) Changes of the phosphorylated AQP2 (S256) expression relative to total AQP2 were examined by semiquantitative immunoblotting (n = 4 in each group). n, number of samples which were prepared from each well.

As shown in Fig. 4, phosphorylated AQP2 expression (S256) was significantly increased by the treatment of synthesized dDAVP (Syn., Peptron Co., Ltd, Daejeon, Korea) and dDAVP-9r peptides as well as purchased dDAVP (Sig., Sigma. St. Louis, MO) for 3 min. Importantly, siRNA/dDAVP-9r polyplex (1∶10 mole ratio) also stimulated AQP2 phosphorylation (S256) in IMCD tubule suspension. The results demonstrate that synthesized dDAVP-9r peptides and siRNA/dDAVP-9r polyplex could bind to V2R and stimulate V2R signaling pathways to phosphorylate AQP2 at S256. This could also induce the endocytosis of V2R.

### Knockdown of AQP2 via V2R-mediated siRNA Delivery Using AQP2-siRNA/dDAVP-9r Polyplex

**Figure 5:**
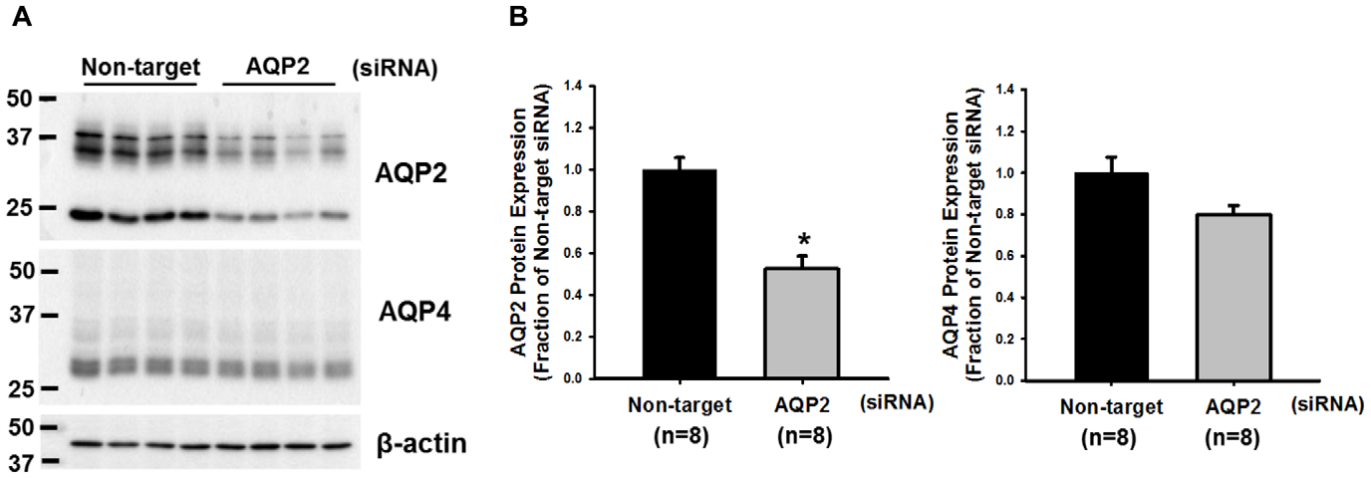
Semiquantitative immunoblotting of AQP2 and AQP4 expression in primary cultured IMCD cells of rat kidney treated by AQP2-siRNA/dDAVP-9r polyplex. **A**) The siRNA/dDAVP-9r polyplex (1∶10) containing 200 pmol of AQP2-siRNA was incubated in the IMCD tubule suspension for 2 h. After incubation, the cells were cultured at 12-well plate for 72 h, and then AQP protein expression was analyzed by semiquantitative immunoblotting. **B**) Protein abundance of AQP2 and AQP4 in IMCD cells treated with the polyplex containing either non-target-siRNA or AQP2-siRNA was evaluated by semiquantitative immunoblotting (n = 8 in each group). *P<0.05 compared with the treatment with non-target-siRNA; n, number of samples which were prepared from each well.

Knockdown of AQP expression *via* V2R-mediated siRNA delivery was investigated in the IMCD cells of rat kidney. For delivering siRNA against AQP2, siRNA/dDAVP-9r polyplex (1∶10 mole ratio) was incubated in the IMCD tubule suspension for 2 h, and then IMCD tubule suspension was seeded at the 12-well plate and cultured for 72 h. The changes of AQP2 protein abundance was analyzed by semiquantitative immunoblotting. AQP2 protein abundance was significantly decreased in the primary cultured IMCD cells treated by AQP2-siRNA/dDAVP-9r polyplex (53±7%, *P*<0.05), compared with the cells treated by non-target-siRNA/dDAVP-9r polyplex (Fig. 5A and B). In contrast, AQP4 protein abundance was unchanged (80±5%, not significant (n.s.)) in the IMCD cells treated by AQP2-siRNA/dDAVP-9r polyplex (Fig. 5A and B), indicating the specificity of AQP2-siRNA/dDAVP-9r polyplex. The observed decrease of AQP2 protein abundance indicates that siRNA could be successfully transduced by dDAVP-9r peptide carrier through V2R interaction.

## Discussion

For the cell-type specific delivery of siRNA, we introduced a novel targeting delivery system using a specific ligand to the hormonal receptor expressed in the target cells. Recent studies suggested that internalization of the receptor proteins after interacting with specific ligands could facilitate the delivery of siRNA into the target cells *via* receptor-mediated siRNA transduction [3], [17], [18], [19].

In the kidney, it has been established that anti-diuretic hormone vasopressin induces internalization of V2R by clathrin-mediated endocytosis [6], [7], [9], [10]. To facilitate siRNA-mediated knockdown of the targeted protein *via* V2R internalization, we designed dDAVP-conjugated peptide carrier for V2R-mediated siRNA delivery in the V2R-expressing IMCD cells. Prediction for the structure of the synthetic peptide carrier conjugated with dDAVP showed two separated regions (i.e., ligand domain to V2R and siRNA carrying domain) bisected with a spacer of 4 glycines. The nine-arginine oligopeptide, which is α-helical form, has been known as the arginine-rich cell-penetrating peptide (CPP) for delivering oligonucleotides into the cells [20], [21], [22]. This synthetic peptide carrier made a stable polyplex with siRNA, i.e., siRNA/dDAVP-9r polyplex at the mole ratio over 1∶10 (siRNA:dDAVP-9r). Importantly, the polyplex induces AQP2 phosphorylation at serine 256 (PKA phosphorylation consensus site) in IMCD cells, indicating that the polyplex could bind to V2R and stimulates V2R. Moreover, the polyplex delivered the FITC-labeled siRNA into the V2R-expressing MDCK cells and LLC-PK1 cells, but not into the V2R-negative Cos-7 cells. This finding indicates that dDAVP-conjugated peptide carrier could successfully deliver siRNA into the V2R-expressing cells via V2R-mediated siRNA transduction. However, it should be noted that polyplex of 1∶40 mole ratio possesses highly positive surface charge, leading to the non-specific interaction with negatively charged plasma membrane, as demonstrated in previous studies [13], [14].

Although the mechanisms of siRNA release from peptide carriers inside the cells have not been clearly elucidated, there are several hypotheses to understand the process of siRNA release. Bartz *et al.* reported that siRNA delivered by amphipathic peptide was escaped from endosomes in pH-dependent manner [23]. Another hypothesis for the mechanism of siRNA release could be achieved by the pore forming ability of arginine-rich peptides [24]. Especially, nine-arginine peptides are known to disturb the stability of the plasma membrane significantly at low pH level [24]. However, further studies are needed to understand the underlying mechanisms of endosomal escape of siRNA from peptide carriers inside the cells.

The stability of nano-particle is a critical obstacle for the application of drug delivery. We demonstrated that the siRNA/dDAVP-9r polyplex was stable in response to the wide range of different osmolalities, pH levels, or to the RNases. Particularly, at the mole ratios of 1∶10 and 1∶40 (siRNA:dDAVP-9r), the siRNA/dDAVP-9r polyplex showed a stability against RNase A. Since siRNA and nine arginine motif of the carrier forms the hydrophobic core by electrostatic interaction [25], [26], siRNA might be protected from RNases which exist in the intracellular vesicles during endocytosis or in the serum [27]. In the process of internalization of siRNA/dDAVP-9r polyplex into the cells, several factors need to be considered, including the size of the polyplex. The adequate size of the polyplex containing ligands for the receptor-mediated internalization is unknown, but it may be dependent on the cell types or the endocytotic pathways. In the case of clathrin-mediated endocytosis, however, it has been reported that particle size might be approximately 200 nm for efficient internalization [28]. Rejman *et al.* [28] showed that microspheres with a diameter less than 200 nm was able to be transduced into the cells by clathrin-mediated endocytosis and delivered to the lysosomes. In contrast, microspheres with a diameter of 500 nm were able to be internalized by caveolae-mediated endocytosis [28]. Since V2R is mainly internalized by clathrin-mediated internalization, the optimal size of the siRNA/dDAVP-9r polyplex might be less than 200 nm. Another important factor required for application of siRNA/peptide polyplex is the capacity of their binding to the receptors. In the present study, phosphorylation of AQP2 (S256) in rat kidney IMCD cells was significantly enhanced by dDAVP-9r peptide as well as siRNA/dDAVP-9r polyplex. The results indicate that siRNA/dDAVP-9r polyplex could directly interact with V2R of IMCD cells and stimulate the receptors. After interaction between V2R and the polyplex, the V2R internalization could be followed, as indicated by the observed uptake of FITC-conjugated siRNA into the cells. Lastly, AQP2-siRNA/dDAVP-9r polyplex effectively delivered siRNA into the IMCD cells, resulting in the significant decrease of protein abundance of AQP2. In contrast, AQP4 protein abundance was unchanged, indicating the specificity of AQP2-siRNA/dDAVP-9r. Since dDAVP could induce V2R internalization and decrease of V2R abundance through lysosomal degradation [9], it may be possible that this contributes to the decrease AQP2 abundance, irrespective of siRNA action. However, in this study, non-target-siRNA/dDAVP-9r was also treated to the control IMCD cells in parallel with AQP2-siRNA/dDAVP-9r treatment, and the results demonstrated the significant decrease of AQP2 abundance selectively in the IMCD cells treated by AQP2-siRNA/dDAVP-9r. Therefore, it is unlikely that dDAVP-induced downregulation of the V2R contributes to the decrease of AQP2 abundance in the IMCD cells.

*In vivo* application of siRNA/dDAVP-9r polyplex directly to rats is potentially possible, since the siRNA/dDAVP-9r polyplex was stable in the diverse environments, e.g., osmolality, pH, or RNases. However, required doses of siRNA to induce knockdown of target protein expression *in vivo* is unknown, and the effective route of administration needs to be determined. Moreover, the effects of dDAVP on urine concentration could be developed, if the ratio of dDAVP-9r peptide to siRNA is high.

In summary, we, for the first time to our knowledge, introduced a novel method of V2R-mediated siRNA delivery in the kidney collecting duct cells using a specific peptide ligand to V2R. siRNA/dDAVP-9r polyplex effectively delivered siRNA into the primary cultured IMCD cells *in vitro*, resulting in the significant decrease of protein abundance of AQPs. This method could be exploited to deliver siRNAs to regulate abnormal expression of specific target proteins *in vivo*, potentially associated with disease conditions in the kidney tubule cells expressing V2R, e.g., collecting duct cells and thick ascending limb cells.

## Materials and Methods

### Ethics Statement

This study was conducted in conformity with the principles of the Declaration of Helsinki. Animal experiments were approved by the Committee on the Ethics of Animal Experiments of the Kyungpook National University (Approval Number: KNU 2011-47).

### siRNAs and Peptide

All siRNAs were purchased from Dharmacon (Chicago, IL) and dDAVP was purchased from Sigma (V1005, St. Louis, MO). Synthetic dDAVP (Mercaptopropionyl-YFQNCPdRG-NH2) and dDAVP-9r (Mercaptopropionyl-YFQNCPdRGGGGGRRRRRRRRR-NH2) peptides were synthesized and purified using high-performance liquid chromatography (Peptron Co., Ltd, Daejeon, Korea). The nine- arginine residue in the C-terminus was D-form of arginine.

### Prediction of dDAVP-9r Peptide Structure

The sequence of dDAVP-9r peptide was submitted to PEP-FOLD server [29], [30], [31] for peptide folding including PSIPRED prediction [32]. PEP-FOLD is a de novo approach aimed at predicting peptide structures from amino acid sequences. Since PEP-FOLD is not able to treat linear peptides with D-amino acids, L-arginine was used for modeling instead of D-arginine. N-terminal modification with mercaptopropionic acid was also excluded from structure calculation.

### Electrophoretic Mobility Shift Assay

The siRNA/dDAVP-9r polyplex was prepared at different mole ratios ranging from 1∶0.1 to 1∶10 (siRNA:dDAVP-9r). siRNA (100 pmol) and dDAVP-9r were incubated in aqueous phase phosphate-buffered saline (PBS 20 µl, pH 7.4) for 15 min at room temperature (RT) for formation of polyplex. The size and size distribution of polyplex were measured by using dynamic light scattering of Zeta-potential & Particle size analyzer (ELS-Z, Otzuka Electronics, Japan). The mixtures were electrophoresed on 2% agarose gel containing ethidium bromide (EtBr). To test stability of the siRNA/dDAVP-9r polyplex against RNAase A, the polyplex containing 100 pmol of siRNA at 1∶10 and 1∶40 mole ratios of siRNA to peptides were incubated in the absence or the presence of RNase A (0.2 µg) for 1 h or 6 h at 37°C. After incubation, 2 µl of 2% SDS solution was added and the sample was loaded on 2% agarose gel containing EtBr. siRNA/dDAVP-9r polyplex was also incubated in various osmolalities produced by NaCl and urea ranging from 0–1,200 mOsm/KgH_2_O for 60 min. Concentrations of NaCl and urea to generate different osmolalities were as follows: 0 mOsm/KgH_2_O (distilled H_2_O), 100 mOsm/KgH_2_O (diluted PBS in the distilled H_2_O), 300 mOsm/KgH_2_O (PBS), 600 mOsm/KgH_2_O (PBS with 150 mM NaCl and 100 mM Urea), and 1,200 mOsm/KgH_2_O (PBS with 400 mM NaCl and 300 mM Urea). In addition, to test the sensitivity of polyplex to pH, siRNA/dDAVP-9r polyplex was incubated for 60 min in various pH levels (5.5–8.0) adjusted with HCl in PBS. The mixture was incubated with or without 0.2% SDS and electrophoresed on 2% agarose gel containing EtBr.

### Cellular Transduction of Fluorescein-labeled siRNA Polyplex in V2R-expressing Cells

FITC-siRNA (100 pmol) and peptides were incubated for 15 min in 20 µl PBS. Cells were incubated with FITC-siRNA/dDAVP-9r polyplex (siRNA:peptide = 1∶10 or 1∶40 mole ratio) for 60 min at 37°C. Moreover, FITC-siRNA or FITC-siRNA/9r peptides (siRNA:peptide = 1∶10) were also incubated for 60 min at 37°C, respectively. Binding of the polyplex containing FITC-siRNA into the MDCK cells (cat. CCL-34, ATCC), LLC-PK1 cells (cat. CL-101, ATCC), and Cos-7 cells (AC28806, KCTC) cells was examined by flow cytometry. After incubation for 60 min, cells were trypsinized and suspensed in PBS. For each determination, the FITC intensity of 10,000 cells was measured on the FL1 channel using a FACS Calibur flow cytometer (Becton-Dickinson, San Jose, CA). Cytometric data was analyzed using CELLQuest software. To access whether siRNA/dDAVP-9r polyplex was internalized into the cells after binding to V2R, subcellular localization was determined using laser scanning confocal microscope (Zeiss LSM 5 EXCITER, Jena, Germany) with a ×63 (NA 1.4) objective lens (Zeiss). Digital images were collected and analyzed using the Zeiss Aim Image Examiner program. MDCK cells at 8-well chamber glass slides (Lab-Tek II, Nunc, Inc., Rochester, NY) were incubated with the polyplex or FITC-siRNA for 60 min at 37°C, and the cells were fixed with 2.5% paraformaldehyde (PFA). The nucleus was stained with DAPI for 3 min.

### Binding Assay of siRNA/dDAVP-9r Polyplex to V2R in Rat IMCD Tubule Suspension

To investigate whether the polyplex containing peptide carriers could stimulate V2R, phosphorylation of AQP2, which is the target protein of vasopressin, was analyzed by immunoblot analysis. Rat IMCD tubule suspension were prepared as a procedure described previously [33] with few modification. The siRNA/dDAVP-9r polyplex was prepared at the mole ratio of 1∶10 (2 nmol of dDAVP-9r). dDAVP (10^−8^ M) alone or siRNA/dDAVP-9r polyplex were added to 100 µL of IMCD tubule suspension and incubated 3 min at RT. After incubation, IMCD tubules were lysed with RIPA buffer (10 mM Tris-HCl, 0.15 M NaCl, 1% NP-40, 1% Na-deoxycholate, 0.5% SDS, 0.02% sodium azide, 1 mM EDTA, pH 7.4) containing protease and phosphatase inhibitors (0.4 µg/ml leupeptin, 0.1 mg/ml pefabloc, 1 mM Na_3_VO_4_, 25 mM NaF, and 0.1 µM okadaic acid).

### Protein Knockdown Assay Using siRNA/dDAVP-9r Polyplex in Primary Cultured Rat Kidney IMCD Cells

IMCD tubule suspensions were incubated with siRNA/dDAVP-9r polyplex containing siRNA (200 pmol) for 2 h at 37°C with gentle shaking. Then, tubules were seeded on the 12-well plate and incubated for 72 h at 37°C. After incubation, the cells were lysed by RIPA buffer containing protease and phosphatase inhibitors for semiquantitative immunoblotting.

### Semiquantitative Immunoblotting

Immunoblot analysis was performed as previously described [34]. Total protein concentrations were measured by BCA assay kit (Pierce, Rockford, IL) and all samples were adjusted with RIPA buffer to reach the identical final protein concentrations and solubilized at 65°C for 15 min in Laemmli sample buffer, and then stored at 4°C. SDS-PAGE was performed on 12% polyacrylamide gel, as previously described [34], [35]. The densitometry values for each protein were corrected by densitometry of β-actin and were normalized to facilitate comparisons.

### Statistical Analysis

Values are presented as means ± SE. Comparisons between two groups were made by unpaired t-test. Comparisions more than two groups were made by one-way ANOVA followed by Tukey HSD’s multiple-comparisons test. Multiple-comparisons tests were only applied when a significant difference was determined in the ANOVA (*P*<0.05). *P* values <0.05 were considered significant.
